# The psychological impact of COVID-19 and lockdown measures among a sample of italian patients with eating disorders: A longitudinal study

**DOI:** 10.1192/j.eurpsy.2021.273

**Published:** 2021-08-13

**Authors:** V. Nisticò, S. Bertelli, A. Priori, O. Gambini, B. Demartini

**Affiliations:** 1 “aldo Ravelli” Research Center For Neurotechnology And Experimental Brain Therapeutics, Università degli Studi di Milano, Milano, Italy; 2 Dipartimento Di Scienze Della Salute, Università degli Studi di Milano, Milano, Italy; 3 Unità Di Psichiatria Ii, ASST Santi Paolo e Carlo, Presidio San Paolo, Milano, Italy; 4 Nutrimente Onlus, Nutrimente Onlus, Milan, Italy; 5 Iii Clinica Neurologica, ASST Santi Paolo e Carlo, Presidio San Paolo, Milano, Italy

**Keywords:** Anxiety, Depression, COVID-19, eating disorders

## Abstract

**Introduction:**

COVID-19 pandemic and lockdown greatly impact on mental health, especially on individuals with pre-existing psychiatric conditions.

**Objectives:**

To explore the prevalence of specific psychiatric symptoms across a sample of patients with Eating Disorder (ED), compared to a group of healthy controls (HC), during the lockdown period in Italy, and to assess whether patients’ symptoms improved, persisted or worsened with the easing of the lockdown measures.

**Methods:**

Study 1: 59 ED patients and 43 HC were recruited and completed, at the beginning of May 2020(t0), an online survey including: the Depression, Anxiety and Stress Scale – 21 items (DASS-21), the Impact of Event Scale-Revised (IES-R), the Perceived Stress Scale (PSS), and few ad-hoc questions extracted from the Eating Disorder Examination Questionnaire (EDE-Q). Study 2: 40 ED patients from Study 1 completed the same survey two months after t0 (t1).

**Results:**

Study 1: ED patients scored significantly higher than HC at the DASS-21 (Total Score and subscales), the IES-R (Total Score and subscales) and the PSS. Moreover, they showed higher distress specifically related to food and their body. Study 2: at t1, levels of stress, anxiety and depression were not different than at t0, but symptoms related to post-traumatic stress disorder (PTSD) improved, together with patients’ reported level of psychological wellbeing and specific ED symptomatology.

**Conclusions:**

During lockdown, ED patients presented significantly higher levels of stress, anxiety, depression, PTSD-related symptoms, and ED-related symptoms than HC. With the easing of lockdown, PTSD-related and ED-related symptoms ameliorated, but high levels of stress, anxiety and depression persisted.

**Disclosure:**

No significant relationships.

